# Inhibition of Corneal Neovascularization by Subconjunctival Injection of Fc-Endostatin, a Novel Inhibitor of Angiogenesis

**DOI:** 10.1155/2015/137136

**Published:** 2015-09-28

**Authors:** Junko Yoshida, Robert T. Wicks, Andrea I. Zambrano, Betty M. Tyler, Kashi Javaherian, Rachel Grossman, Yassine J. Daoud, Peter Gehlbach, Henry Brem, Walter J. Stark

**Affiliations:** ^1^Wilmer Eye Institute, Johns Hopkins University School of Medicine, Baltimore, MD 21287, USA; ^2^Department of Neurosurgery, Johns Hopkins University School of Medicine, Baltimore, MD 21231, USA; ^3^Center of Cancer Systems Biology, St. Elizabeth's Medical Center, Tufts University School of Medicine, Boston, MA 02111, USA; ^4^Departments of Oncology and Biomedical Engineering, Johns Hopkins University School of Medicine, Baltimore, MD 21205, USA

## Abstract

We assessed the antiangiogenic effects of subconjunctival injection of Fc-endostatin (FcE) using a human vascular endothelial growth factor-induced rabbit corneal neovascularization model. Angiogenesis was induced in rabbit corneas through intrastromal implantations of VEGF polymer implanted 2 mm from the limbus. NZW rabbits were separated into groups receiving twice weekly subconjunctival injections of either saline; 25 mg/mL bevacizumab; 2 mg/mL FcE; or 20 mg/mL FcE. Corneas were digitally imaged at 5 time points. An angiogenesis index (AI) was calculated (vessel length (mm) × vessel number score) for each observation. All treatment groups showed a significant decrease in the vessel length and AI compared to saline on all observation days (*P* < 0.001). By day 15, FcE 2 inhibited angiogenesis significantly better than FcE 20 (*P* < 0.01). There was no significant difference between FcE 2 and BV, although the values trended towards significantly increased inhibition by BV. BV was a significantly better inhibitor than FcE 20 by day 8 (*P* < 0.01). FcE was safe and significantly inhibited new vessel growth in a rabbit corneal neovascularization model. Lower concentration FcE 2 exhibited better inhibition than FcE 20, consistent with previous FcE studies referencing a biphasic dose-response curve. Additional studies are necessary to further elucidate the efficacy and clinical potential of this novel angiogenesis inhibitor.

## 1. Background

To maintain transparency, the cornea is an avascular tissue. Infectious and inflammatory processes, however, can induce new vessel growth causing corneal neovascularization, which leads to scarring, edema, and blindness [1]. Corneal neovascularization affects an estimated 4.1% of all patients presenting to general ophthalmology offices in the US [2]. It also contributes to worsening prognosis after penetrating keratoplasty (PK). A meta-analysis has shown a significantly higher risk of corneal graft rejection with increased number of corneal quadrants affected by neovascularization prior to keratoplasty [3].

Corneal neovascularization is thought to occur through the imbalance of angiogenic and antiangiogenic protein factors. Vascularized human corneas have been shown to have significant upregulation of vascular endothelial growth factor (VEGF), matrix metalloproteinases (MMP), and basic fibroblastic growth factor (bFGF) [4]. Bevacizumab (BV) (Avastin, Genentech, Inc., San Francisco, CA) is a monoclonal antibody that targets human VEGF (VEGF) and has been studied as a potentially potent therapy for corneal neovascularization. Antiangiogenic monotherapy with a drug such as BV, which targets VEGF alone, however, may not provide the best long-term therapy since inhibition of one antiangiogenic factor can result in the upregulation of others leading to acquired drug resistance [5]. In addition, lymphangiogenesis has been identified as an important process involved in the pathogenesis of corneal graft rejection. Endostatin, in contrast to bevacizumab, has been identified as a potent inhibitor of both neovascularization and lymphangiogenesis [6–8].

Another potential deficiency of BV therapy is its association with systemic side effects seen during trials of its use in age-related macular degeneration, including bleeding, hypertension, and stroke [9–12]. Thus, an angiogenesis inhibitor that blocks multiple promoters of angiogenesis with few systemic side effects is desired.

Endostatin promised such potential. As a 20 kDa fragment of collagen XVIII, it modifies 12% of the human genome in order to downregulate angiogenesis with few systemic side effects [13–15]. By affecting multiple angiogenic pathways simultaneously [16], endostatin may allow for a much lower possibility of drug resistance. In contrast to the noted hypertension found with BV use in human studies [17, 18], endostatin was noted to decrease systolic blood pressure by almost 10 mmHg when dosed daily in animal studies [19]. Endostatin was initially studied in preclinical models of corneal neovascularization and tumor models [20, 21]. Despite high expectations, human endostatin had a limited response in phase I and II human cancer clinical trials largely due to two deficiencies. First, the half-life of endostatin in circulation was only 42.3 minutes. Second, approximately 50% of the recombinant human endostatin used in the clinical trials lacked four amino acids at the NH_2_ terminus, which resulted in a nonfunctional molecule due to an inability to bind zinc [22].

In order to engineer a more stable form of endostatin, Fc-endostatin (FcE) was developed by fusing endostatin to the Fc region of an IgG molecule [22]. The presence of the Fc portion increases the half-life to greater than one week and conserves the molecule's zinc affinity. This increase in half-life is similar to the role of the Fc domain in BV, which increases the molecule's half-life to weeks rather than hours [23]. FcE was recently shown to be effective in a rodent model of high-grade glioma using various delivery methodologies [24].

We set out to evaluate the safety and antiangiogenic efficacy of subconjunctival injection of FcE using a rabbit VEGF-induced corneal neovascularization model. This paper is the first published assessment of FcE as a potential therapy for corneal neovascularization.

## 2. Methods

### 2.1. Animals

Eight male New Zealand White rabbits, each weighing 4.5 to 6.5 kg (Robinson Industry Farms, Mocksville, NC) were used. Care and treatment of all rabbits were in strict agreement with the Association for Research in Vision and Ophthalmology (ARVO) Statement for the Use of Animals in Ophthalmic and Vision Research and with the approval of the Johns Hopkins University Animal Care and Use Committee. Rabbits were housed in standard animal facilities, one animal per cage, and given free access to food and Baltimore City water.

### 2.2. Anesthesia

For intracorneal implantations and subsequent operative microscope examinations the animals were anesthetized with a mixture of xylazine 10 mg/kg (Butler Schein, Dublin, OH) and ketamine 2 mg/kg (Butler Schein, Dublin, OH). The ocular surface was anesthetized with topical 0.5% proparacaine hydrochloride (Alcaine, Alcon, Fort Worth, TX). Pain control was provided, as needed, with subcutaneous injection of buprenorphine HCL 0.1 mg/kg (Bedford Labs, Bedford, OH).

### 2.3. VEGF Polymer Preparation

Ethylene-vinyl acetate copolymer (40% vinyl acetate by weight, Elvax 40P) (Du Pont Co., Wilmington, DE) was prepared as previously described [19]. Briefly, human VEGF (Peprotech, Rocky Hill, NJ) was incorporated into the ethylene-vinyl acetate copolymer (EVAc) matrix. EVAc (130 mg) was dissolved in methylene chloride (1.8 mL) and VEGF (20 *μ*g) was added. The mixture was poured into cylindrical glass molds measuring 5 mm × 220 mm and placed in −20°C for 48 hours and the methylene chloride was allowed to passively evaporate. The VEGF polymer was then cut into uniform pellets of size 1 mm × 1 mm × 0.5 mm. The VEGF amount per implanted polymer pellet totaled 770 ng.

### 2.4. VEGF-Induced Corneal Neovascularization Model

**Figure 1:**
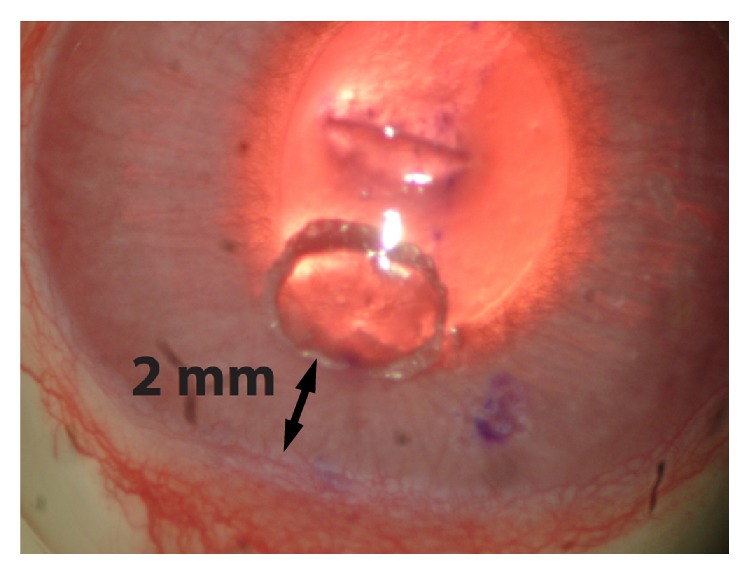
VEGF-induced corneal neovascularization model. Each human vascular endothelial growth factor (VEGF) polymer was inserted into surgically created corneal micropockets. Two micropockets, at 6 and 12 o'clock, were made from the midline incision. One VEGF polymer was placed at the end of each micropocket at a distance of 2 mm from the limbus.

We use a corneal angiogenesis model as previously described by Sefton et al. [25]. Rabbits were placed under general anesthesia and topical 0.5% proparacaine hydrochloride was applied to the corneal surface. 5% povidone-iodine was applied on the ocular surface for antisepsis. At the center of the cornea, a 2 mm horizontal incision was placed to the midstromal level using the bevel of a 16 gauge needle (BD, Franklin Lakes, NJ). Two micropockets were made from the incision towards 6 and 12 o'clock by dissecting the corneal stroma with an iris spatula (Figure 1). One VEGF polymer was placed at the end of each micropocket 2 mm apart from the limbus. A total of 32 VEGF polymer pellets were placed (2 pellets per cornea, one at 6 o'clock and one at 12 o'clock). The surgery day was referred to as day 0.

Rabbits were separated into 4 groups (8 pellets per group). Each eye received a subconjunctival injection, one half of each dose was injected at 6 and 12 o'clock in proximity to each VEGF polymer, with either 0.125 mL of 0.9% normal saline (NS), 0.1 mL of 25 mg/mL BV, 0.125 mL of 20 mg/mL human FcE (FcE 20) (Bio X Cell, West Lebanon, New Hampshire), or 0.125 mL of 2 mg/mL human FcE (FcE 2). A volume of 0.125 mL of FcE was used in order to approximate the mg concentration of BV 25 mg/mL. Subconjunctival injections were performed twice weekly starting on the day of surgery: days 0, 5, 8, and 12.

### 2.5. Corneal Neovascularization Evaluation

Corneal neovascularization was assessed via operative microscopy (Carl Zeiss, Germany) on days 0, 5, 8, 12, and 15. The corneas were digitally imaged, and the images were analyzed using ImageJ software (NIH.gov). To have a single standardized value that incorporated both vessel length and number of vessels, we calculated an angiogenesis index (AI), as previously described by Tamargo et al. [26]. Briefly, the number of new vessels associated with each VEGF polymer was given a numbered score: 0 = no vessels, 1 = <10 vessels, 2 = ≥10 vessels with a visible iris, and 3 = ≥10 vessels with no visible iris. Neovessel length was assessed as distance from the limbus to the leading edge of the new vessels in millimeters (mm). AI was then calculated as

(1) $$\begin{array}{c} AI=vessel\;length\;\left(mm\right)\times vessel\;number\;score. \end{array}$$

Given that the greatest vessel length achievable was 2 mm (distance to the VEGF polymer from the limbus) and the highest vessel number score was 3, the AI ranged from 0 to 6.

### 2.6. Histological Evaluation of Corneas

All rabbits were sacrificed after the last observation on day 15. Both eyes of all rabbits were enucleated and placed in 10% formalin. After fixation in formalin, corneas were removed and cut in half at the midline, one VEGF polymer pellet in each half. All cornea halves were embedded in paraffin with the cornea-sclera border facing down, sectioned, and stained with hematoxylin and eosin (H&E). The extent of neovascularization was analyzed and photographed using digital, light microscopy (BX41, Olympus, Japan; Diagnostic Instruments, Inc., Sterling Heights, MI).

### 2.7. Statistical Analysis

Total sample size was 32 with measurements of neovascularization taken on days 5, 8, 12, and 15. Vessel length and AI values recorded throughout the observation period were analyzed for statistical significance using a repeated measures 2-way analysis of variance (2-way ANOVA). Post hoc analysis was performed using the Bonferroni test to correct for multiple comparisons (Graphpad Prism 5.0, CA).

## 3. Results

### 3.1. Analysis of Corneal Neovascularization

**Figure 2:**
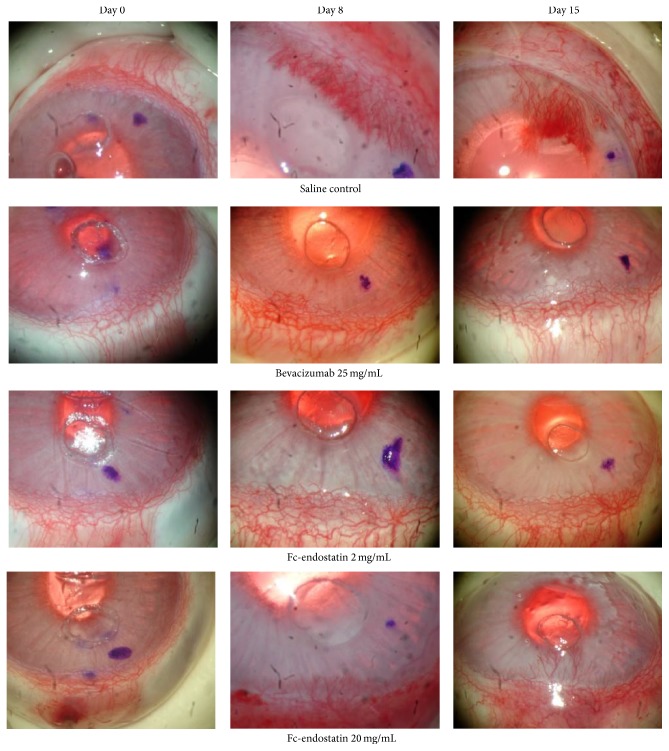
Representative photos of each treatment group on days 0, 8, and 15 after implantation of human vascular endothelial growth factor (VEGF) polymer within the corneal stroma micropocket. Treatment drugs were delivered via subconjunctival injection on days 0, 5, 8, 12, and 15. No statistical difference was noted between bevacizumab (BV) and Fc-endostatin 2 mg/mL (FcE 2). BV was found to significantly decrease neovascularization when compared with Fc-endostatin 20 mg/mL (FcE 20) by day 8. FcE 2 was found to significantly decrease neovascularization compared with FcE 20 by day 15.

**Figure 3:**
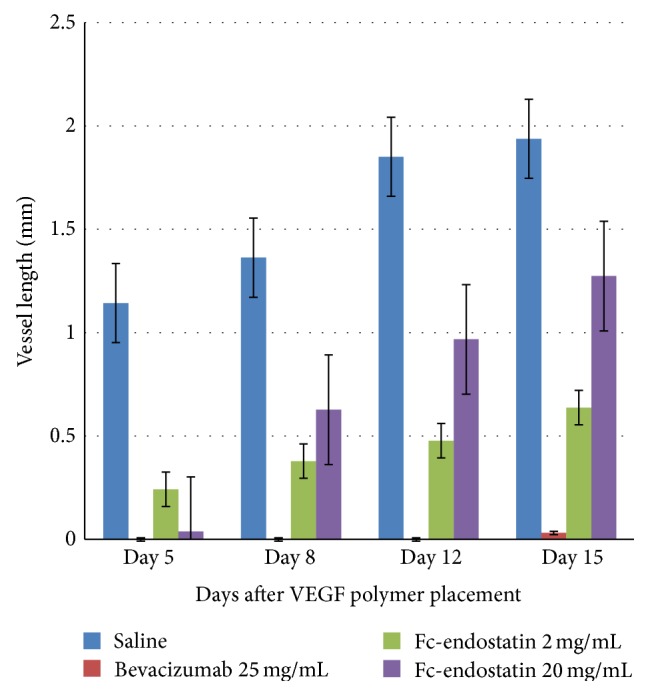
Inhibition of human vascular endothelial growth factor- (VEGF-) induced corneal neovascularization in rabbit. Mean vessel length ± standard error of the mean (SEM) of new vessels in control and treatment groups (BV: bevacizumab; FcE: Fc-endostatin).

Neovascularization was analyzed with respect to both vessel length and AI. The representative images taken on days 0, 8, and 15 before and after treatment with BV and FcE are shown in Figure 2.  Figure 3 displays the mean vessel length for each treatment group. While NS control developed corneal vessels nearly 2 mm length, all treatment groups had a statistically significant decrease in vessel length compared to NS on all observation days (*P* < 0.001). FcE 2 significantly decreased vessel length more than FcE 20 on days 12 and 15 (*P* < 0.05). BV inhibited vessel length significantly more than FcE 2 on days 12 and 15 (*P* < 0.05). BV decreased vessel length significantly more than FcE 20 on days 8, 12, and 15 (*P* < 0.01).

**Figure 4:**
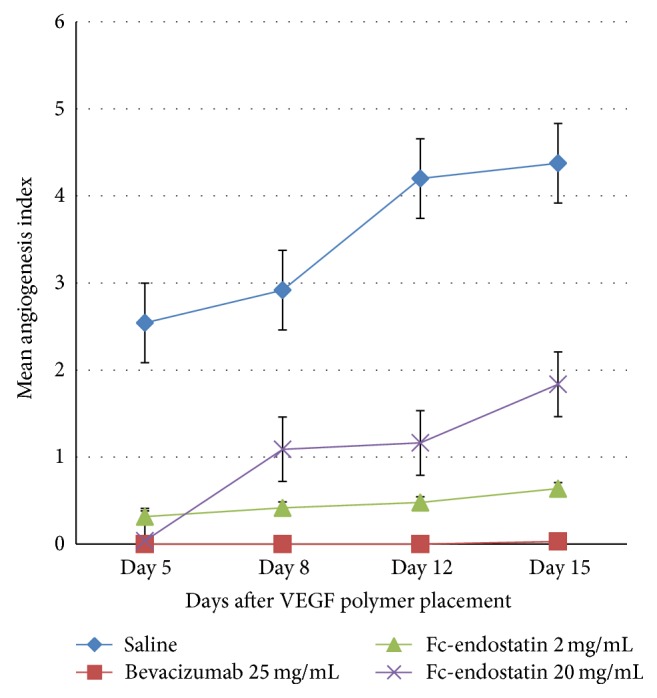
Inhibition of human vascular endothelial growth factor- (VEGF-) induced corneal neovascularization in rabbit. Mean angiogenesis index (AI) ± standard error of the mean (SEM) of new vessels in control and treatment groups (BV: bevacizumab; FcE: Fc-endostatin).

Corneal neovascularization was then analyzed by calculating AI (Figure 4). AI provided a more comprehensive analysis of the neovascularization as it reflects a more direct assessment of neovessel density. All treatment groups showed a significant decrease in the AI compared to NS on all observation days (*P* < 0.001). FcE 2, when compared to NS, decreased AI by a factor of 8.0-fold, 7.0-fold, 8.8-fold, and 6.8-fold on days 5, 8, 12, and 15, respectively. FcE 20, when compared to NS, decreased AI by a factor of 68-fold, 2.7-fold, 3.6-fold, and 2.4-fold at days 5, 8, 12, and 15, respectively. BV decreased AI compared to NS by a factor of 138-fold on day 15, with no vessel growth at days 5, 8, and 12. A significant difference in AI was not found between BV and FcE 2 during the study period, although BV appeared to have a nonsignificant qualitative improvement over FcE 2 during the study period. BV was found to have a significantly smaller AI than FcE 20 on days 8, 12, and 15 (*P* < 0.01). By day 15, AI of FcE 2 was significantly smaller than FcE 20 (*P* < 0.01). No obvious side effects were observed topically or systemically from either FcE or BV.

### 3.2. Analysis of Cornea Histology

**Figure 5:**
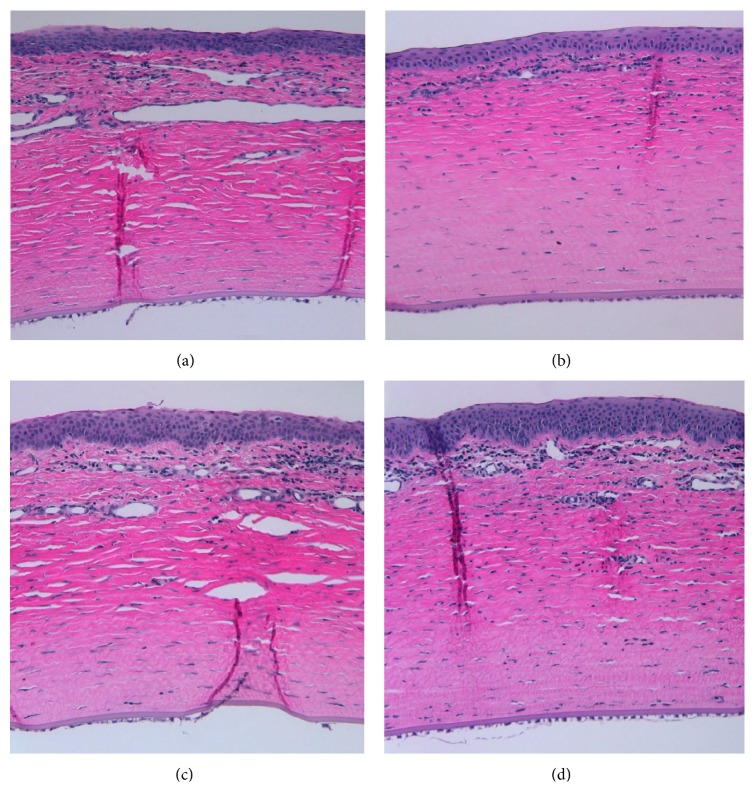
Corneal histology. On day 15, all eyes were enucleated and fixed in formalin. After fixation in formalin, corneas were removed, embedded in paraffin, and stained with hematoxylin and eosin (H&E). Pictured are H&E stained slices taken at the cornea-sclera border. The cornea epithelial surface is facing up. (a) Normal saline (group); (b) bevacizumab 25 mg/mL; (c) Fc-endostatin 2 mg/mL; and (d) Fc-endostatin 20 mg/mL.

The cornea sections within the NS group showed the greatest corneal neovascularization with a relative increase in both vessel number and vessel circumference when compared with the treatment groups (Figure 5). New vessels were observed within both the superficial and deep stroma. Corneas from the BV treated group had the fewest number of new vessels. The neovascularization present in the BV group was primarily isolated to the superficial stroma and showed a relative decrease in circumference. FcE corneal sections revealed decreased neovascularization, with decreased vessel circumference, when compared with NS but a greater amount than corneas within the BV group. There was no notable histological difference between the neovascularization that occurred in the FcE 2 group and the FcE 20 group.

## 4. Discussion

Subconjunctival injection of FcE was found to be well tolerated and to significantly decrease corneal neovascularization in a rabbit model of VEGF-induced neovascularization. Although BV inhibited the corneal neovascularization best in this model, no statistical difference was identified between FcE 2 and BV. FcE 20 had an early decrease in neovascularization noted on day 5, but this benefit was less than that noted by FcE 2 on day 8. Both BV and FcE 2 significantly inhibited corneal neovascularization better than FcE 20. No corneal ulcers, edema, infection, or conjunctival necrosis was noted as a result of either the subconjunctival injection of FcE or BV.

The purpose of this study was as a proof of concept to determine the safety and relative efficacy of FcE in inhibiting corneal neovascularization. We chose to use a VEGF-induced corneal neovascularization model and as a positive control BV, a monoclonal antibody to VEGF, was used. In the short time course of this study, BV inhibited the corneal neovascularization the most, as expected. Human FcE, however, was found to be noninferior to BV, representing an important finding. FcE may be more effective than BV on a multicytokine angiogenesis model given its known multitargeted antiangiogenic effect.

FcE 2 was found to outperform the higher concentration FcE 20 by the completion of the experiment on day 15. Initially, FcE 20 had an impressive decrease in neovascularization on day 5, but this effect had weaned by day 15. This finding had been described by Celik et al., who noted the antitumor activity of endostatin to have a biphasic dose-response curve [22, 27]. Efficacy is found to proportionally increase until an optimal dose is reached with higher doses resulting in decreased response. A biphasic dose-response curve has been noted in studies of other antiangiogenic cytokines such as interferon-*α* (IFN-*α*) [27, 28] and the diabetes medication rosiglitazone, a known tumor cell angiogenesis inhibitor [29]. FcE has also been demonstrated to have a similar pattern of activity [30]. Further dose response analysis will need to be performed within the corneal neovascularization model to fully characterize the dose-response relationship.

Several limitations to the current study should be taken into account. The first limitation comes as a result of the corneal neovascularization model selected that allows for the controlled release of VEGF and does not entirely mimic the natural proangiogenic environment in which corneal neovascularization develops within a damaged cornea. Additional studies utilizing other models such as the mechanical limbal injury-induced corneal neovascularization model [31], alkali-induced corneal neovascularization model [31, 32], and the corneal micropocket tumor implantation model [26] should be considered in future experiments. Secondly, the study was not designed to determine the most optimal dose of FcE or the cause of the biphasic dose response. Additional studies to determine the mechanism of inhibition and establish a complete dose-response curve are warranted. A third limitation of the current study is that long-term efficacy of FcE was not assessed. Further preclinical research would need to be completed prior to pursuing human trials into FcE as a potential novel inhibitor of corneal neovascularization.

## 5. Conclusions

FcE is a novel antiangiogenic compound shown to significantly inhibit new vessel growth in a rabbit corneal neovascularization model. Lower concentration FcE 2 exhibited better inhibition than FcE 20—consistent with previous FcE studies referencing a biphasic dose-response curve. The FcE 2 concentration was found to be noninferior to BV in this VEGF model. Because of its known multitargeted, antiangiogenic properties, FcE holds great promise for future clinical efficacy. Further studies are necessary to elucidate the clinical potential and optimal dosing of this novel inhibitor of corneal neovascularization.

## Abbreviations

VEGF: Human vascular endothelial growth factor
BV: Bevacizumab
FcE: Fc-endostatin
EVAc: Ethylene-vinyl acetate copolymer
FcE 20: 20 mg/mL human FcE
FcE 2: 2 mg/mL human FcE
AI: Angiogenesis index.
